# Microscopic and molecular detection of *Trichomonas vaginalis* in outpatients seeking medical care in Upper Egypt

**DOI:** 10.3389/fmicb.2024.1499270

**Published:** 2024-11-20

**Authors:** Nasser Mohamed Abd El-kareem, Ahmed Kamal Dyab, Nada Oudah Albalawi, Abdalla Abd El Samea, Mohamed Ahmed Ali Taha, Hajar AlQadeeb, Ahmed Gareh, Elham Adel Hiekal, Hind Alzaylaee, Ehab Kotb Elmahallawy

**Affiliations:** ^1^Department of Medical Parasitology, Faculty of Medicine, Al-Azhar University (Assiut Branch), Assiut, Egypt; ^2^Department of Medical Parasitology, Faculty of Medicine, Assiut University, Assiut, Egypt; ^3^Department of Parasitology, School of Veterinary Medicine, Badr University in Assiut, Assiut, Egypt; ^4^Department of Biology, Faculty of Science, Taibah University, Alula, Saudi Arabia; ^5^Department of Medical Laboratory, College of Applied Medical Sciences, Prince Sattam Bin Abdulaziz University, AlKharj, Saudi Arabia; ^6^Department of Parasitology, Faculty of Veterinary Medicine, Aswan University, Aswan, Egypt; ^7^Department of Medical Parasitology, Faculty of Medicine, Al-Azhar University, Cairo, Egypt; ^8^Department of Biology, College of Science, Princess Nourah Bint Abdulrahman University, Riyadh, Saudi Arabia; ^9^Departamento de Sanidad Animal, Grupo de Investigación en Sanidad Animal y Zoonosis (GISAZ), UIC Zoonosis y Enfermedades Emergentes ENZOEM, Universidad de Córdoba, Córdoba, Spain; ^10^Department of Zoonoses, Faculty of Veterinary Medicine, Sohag University, Sohag, Egypt

**Keywords:** *Trichomonas vaginalis*, nested PCR, genotyping, RFLP, Upper Egypt

## Abstract

**Introduction:**

Trichomoniasis remains one of the most significant sexually transmitted disease (STDs) for public health. The disease is caused by parasitic protozoa, *Trichomonas vaginalis* (*T. vaginalis*), which is often underestimated in tropical medicine. Despite its public health importance, the epidemiology and molecular characteristics of trichomoniasis in Egypt remains poorly understood, particularly in the southern part of the country (Upper Egypt). This study targeted exploring the genetic variability of *T. vaginalis* infections in Egyptian women living in Upper Egypt using restriction fragment length polymorphism (RFLP).

**Patient and techniques:**

This cross-sectional study included 150 female patients, who visited the gynaecology and obstetrics outpatient clinics at Sohag General Hospital between 2019 and 2022, exhibiting symptoms of trichomoniasis. Vaginal washout samples were collected from each patient and analyzed using three diagnostic techniques: direct wet mount microscopy, culture on TYM Diamond’s medium, and PCR amplification and Polymerase Chain Reaction-Restriction Fragment Length Polymorphism (PCR-RFLP) targeting the actin gene, which was applied to all 16 samples that tested positive in culture. The PCR-RFLP results were then visualized through agarose gels electrophoresis to detect DNA fragments.

**Results:**

Out of 150 vaginal washout samples, 12 cases (8%) tested positive for *T. vaginalis* trophozoites via direct wet mount microscopy, while 16 samples (10.6%) were positive in culture. Additionally, PCR-RFLP analysis of the 16 culture-positive samples revealed that 13 samples were confirmed positive using this molecular method. The amplified products were digested with the restriction enzyme Hind II, yielding three DNA fragments of 60, 213, and 827 bp, which were then detected by agarose gel electrophoresis. Digestion with RsaI produced five fragments measuring 87, 103/106, 236, and 568 bp, while MseI digestion resulted in three distinct fragments of 204, 315, and 581 bp.

**Conclusion:**

This study provides robust baseline data on the prevalence and microscopic characteristics of *T. vaginalis* in Upper Egypt, while also presenting, for the first time, molecular detection and genotyping and revealed that genotype E is the only prevalent genotype in the region.

## 1 Introduction

Trichomoniasis is a widespread sexually transmitted infection caused by the parasite *Trichomonas vaginalis*, which impacts both men and women. The infection by this parasite typically causes symptoms like vaginal discharge and itching in women, though many individuals may experience no symptoms. The World Health Organization (WHO) estimates that approximately 143 million new cases of trichomoniasis occur annually (WHO, 2016), with prevalence rates varying worldwide due to factors such as geographic location, age, race, community, and diagnostic methods. In 2016, WHO estimated the global prevalence of trichomoniasis at 5.3% in women and 0.6% in men, resulting in a total of 156 million cases (Rowley et al., 2019). Regarding its clinical impact, trichomoniasis is linked to serious reproductive complications, including infertility, ectopic pregnancy, pelvic inflammatory disease, premature rupture of membranes, preterm birth, and low birth weight (Hamouda et al., 2022). It also plays a critical role in the transmission and acquisition of human immunodeficiency virus (HIV) (Alves et al., 2020) and human papillomavirus (Heikal et al., 2023). Taken into consideration, the variation in clinical outcomes, such as differences in virulence, pathogenicity, and drug resistance, highlights the need to connect these phenotypic differences to specific genotypes (Hawksworth et al., 2015). The genome of *T. vaginalis* comprises over 60,000 protein-coding genes within a large 160 Mb genome, predominantly consisting of transposable elements and repetitive sequences (Carlton et al., 2007). The primary cytoskeleton of *T. vaginalis* is composed of actin proteins, which are essential for cellular motility and adhesion (Crucitti et al., 2008). Due to its extensive conservation across the species, actin is a promising target for molecular identification within *T. vaginalis* (Drouin et al., 1995). It should be also stressed that PCR and related techniques are widely used for genetic research on various organisms. The random amplified polymorphic DNA (RAPD) method has been instrumental in linking metronidazole resistance to specific genotypes of *T. vaginalis* (Meade and Carlton, 2013).

Recent advancements in the genetic analysis of *T. vaginalis* isolates revealed a strong link between the organism’s genetic diversity and the wide range of clinical outcomes in trichomoniasis, as well as its disease-related complications (Leitsch, 2016). Several methods of genotyping of the parasite revealed extensive genetic variation among *T. vaginalis* strains (Meade and Carlton, 2013). In this regard, molecular studies consistently identified two predominant genotypes of *T. vaginalis*. Genotype I is believed to be evolutionarily older than genotype II, as it displays greater genetic variation. Interestingly, both genotypes are found in similar proportions globally, although genotype I is more prevalent in South Africa, whereas genotype II is more common in Mexico. Clinically, genotype I tends to be associated with more pathogenic infections, while genotype II has been linked to resistance to metronidazole (van der Veer et al., 2016). Additionally, previous research (Meade et al., 2009) employed PCR-RFLP techniques to reveal the genetic diversity among clinical isolates of *T. vaginalis*. Currently, the PCR-RFLP technique, based on the amplification of the actin gene, is considered a sensitive and reliable method for typing *T. vaginalis* isolates (Tavakoli Oliaee et al., 2017). Studies employing this technique have been conducted in Iran (Orujzadeh et al., 2019), China (Zhang et al., 2018), and Turkey (Demirağ et al., 2017) and showed that genotype E is the most prevalent among twenty *T. vaginalis* isolates from symptomatic females, followed by genotype G. Only one isolate was identified as genotype H, and two isolates were mixed, containing both genotypes. Similarly, Zhang et al. (2018) reported three mixed genotypes and two isolates with E and H genotypes out of 68 isolates. Recent research in Iran identified three genotypes—E, G, and I—in *T. vaginalis* isolates infected with dsRNA viruses (Orujzadeh et al., 2019). Understanding the genetic diversity of *T. vaginalis* populations is crucial for the effective prevention and management of trichomoniasis.

In Egypt, various studies performed in several Egyptian governorates, including Beni Suef, Qalyubia and Cairo governorates, and reported a prevalence rate which is ranged from 3 to 11% (Hamdy and Hamdy, 2018; Hussein et al., 2015; Mahmoud et al., 2015). Earlier efforts to investigate the diversity of *T. vaginalis* in Egypt included isomeric pattern analysis (Salem et al., 1992), serotyping (Salem et al., 1992), immunoblotting (El-Okbi et al., 2005), biological variability studies (Laila et al., 2012), HSP70-RFLP (Hussein et al., 2015), and multilocus sequence typing (Mohamed et al., 2019). These studies concluded that clinical isolates of *T. vaginalis* might exhibit both unique and shared patterns in terms of antigen levels, immunogens, pathogenicity, and metronidazole (MTZ) resistance. However, upon reviewing the available literature, there is a notable lack of data on the molecular characteristics of *T. vaginalis* among women in Upper Egypt. In addition to summarizing previous studies conducted on *T. vaginalis* at the national level (Table 1; references: Hamouda et al., 2022; Hamdy and Hamdy, 2018; Mahmoud et al., 2015; Mohamed et al., 2019; Issa and Shalaby, 2006; Hamed Elsherif and Fatah Youssef, 2013; Khalifa et al., 2018; El Sayed et al., 2010; El-Gayar et al., 2016; Shawaky et al., 2022; Kamal et al., 2018; Hamed et al., 2018; Heikal et al., 2023; Ibrahim et al., 2021; Farouk et al., 2021; Hashem et al., 2024), this study aims to investigate the genetic diversity of *T. vaginalis* in women from Upper Egypt, employing PCR-RFLP methods alongside morphological and microscopic techniques.

**Table 1:** Occurrence and genetic diversity of *T. vaginalis* in women populations in Egypt.

Location	Governorate	Detection method	Infection rate (%)	Pos./Total	Genotype	Reference
Lower Egypt	Cairo	WM	39.13	9/23	–	Issa and Shalaby (2006)
		Culture	69.56	16/23	–	
		PCR	71.42	5//7	–	
		WM	11.90	60/504	–	Hamed Elsherif and Fatah Youssef (2013)
		Culture	23.01	116/504	–	
		PCR	66.00	33/50	–	
		WM	4.0	12/300	–	Khalifa et al. (2018)
		LAT	5.0	50/1000	–	Mahmoud et al. (2015)
		Culture	3.0	30/1000	–	
		GS	1.0	10/1000	–	
		WM	1.0	10/1000	–	
		WM & Culture	4.0	12/300	–	Mohamed et al. (2019)
		PCR	91.7	11/12	–	
	Giza	WM	42.03	124/295		Hashem et al. (2024)
	Dakhalia	WM & GS	30.0	33/110	–	El Sayed et al. (2010)
		WM & GS	8.50	17/200		Hamouda et al. (2022)
		Culture in DM	12.0	24/200	–	
	Ismaillia	WM & Culture in DM	37.69	98/260	–	El-Gayar et al. (2016)
		WM & Culture	36.36	40/110	–	El-Gayar et al. (2016)
	Alexandria	WM	0.58	3/516	–	Shawaky et al. (2022)
						
Upper Egypt	El-Minia	WM	9.66	29/300	–	Kamal et al. (2018)
		Urine	7.33	22/300	–	
		Culture in DM	11.66	35/300	–	
	El Fayoum	WM	24.0	24/100	–	Hamed et al. (2018)
		GS	9.0	09/100	–	
		Field Stain	24.0	24/100	–	
		WM	8.33	8/96	–	Heikal et al. (2023)
		Giemsa	10.41	10/96	–	
		PCR	29.16	28/96	–	
	Beni-Suef	LAT	8.0	8/100	–	Hamdy and Hamdy (2018)
		WM	1.0	1/100	–	
		Giemsa	3.0	3/100	–	
		Culture in DM	6.0	6/100	–	
		WM	6.66	6/90	–	Ibrahim et al. (2021)
		GS	6.66	6/90	–	
		Culture	15.0	9/90	–	
	Aswan	WM	5.0	10/200	–	Farouk et al. (2021)
		GS	7.50	15/200	–	
		Culture	13.50	27/200	–	
		Rapid test	12.50	25/200	–	
		ELISA	15.00	30/200	–	

WM, wet mount; GS, Giemsa stain; DM, Diamond media; PCR, polymerase chain reaction; LAT, latex agglutination test; ELISA, enzyme linked immunosorbent assay; − or ND, not determined.

## 2 Patient and methods

### 2.1 Ethical considerations

The study design was reviewed and approved by the Research Ethics Committee of the Faculty of Medicine at Assiut University, adhering to the Declaration of Helsinki and regulations set forth by the Egyptian Ministry of Higher Education. Informed consent was obtained from each participant after a comprehensive explanation of the study’s objectives. Appropriate treatment was prescribed by a gynaecologist for participants who tested positive for trichomoniasis (Ethical No. 1720024/27-8-2018).

### 2.2 Study population

This observational study involved 150 women suspected of trichomoniasis, attending the obstetrics and gynaecology outpatient clinics at Sohag General Hospital between 2019 and 2022.

### 2.3 Inclusion criteria

Women were included if they were not menstruating and presented with gynecological symptoms suggestive of trichomoniasis, such as vaginal discharge, dyspareunia, dysuria, or vulvar pruritus.

### 2.4 Exclusion criteria

Women were excluded if they had engaged in sexual activity or douching within the past 2 days or had used antiprotozoal or antibiotic treatments in the previous 2 weeks. Menstruating women and virgins were also excluded. Each participant completed a detailed medical history and provided demographic information. After a thorough explanation of the study, all participants signed informed consent forms.

### 2.5 Sample collection

Vaginal washout samples were collected from participants according to the method described by McMillan et al. (1979). A sterile Cusco speculum was inserted into the vagina and adjusted to a 90-degree angle to enhance visibility of the cervix. Subsequently, 5 mL of sterile isotonic PBS solution were injected intravaginally using a needle-free sterile syringe. The fluid was then aspirated from the posterior fornix with a plastic pipette and transferred into sterile tubes. The samples were analyzed using direct wet mount microscopy and cultured on modified Diamond’s medium for the detection of *T. vaginalis*. Additionally, a portion of each sample was aliquoted into 1.5 mL microcentrifuge tubes and stored at −20°C for further PCR analysis.

### 2.6 Examination of vaginal washout

Following sample collection, a direct wet mount microscopic examination was performed using a light microscope with ×10, ×40, and ×100 objectives. This procedure aimed to promptly identify motile *T. vaginalis* trophozoites by observing their characteristic flagellate movement (Radonjic et al., 2006). The modified TYM Diamond culture medium was prepared in the parasitology laboratory of the Faculty of Medicine, Assiut University, following the protocol described by Diamond (1957). The medium consisted of the following ingredients: 24 g of Tryptone (Oxoid, United Kingdom), 12 g of Yeast Extract Powder (Oxoid, United Kingdom), 6 g of Maltose (Arabic Laboratory Equipment Co., Egypt), 1.2 g of L-Cysteine Hydrochloride (WINLAB, Egypt), 0.24 g of L-Ascorbic Acid (Arabic Laboratory Equipment Co., Egypt), and 900 mL of distilled water. The pH was adjusted to 6.3 using the pH meter and by adding 1 N HCL. The medium, contained in a Pyrex tube, was autoclaved at 121°C for 25 min and then allowed to cool. After cooling to 50°C, 100 mL of sterile inactivated horse serum (VACSERA, Dokki, Egypt) and an antibiotic mixture—prepared by adding 2 mL of sterile distilled water to each vial of penicillin and streptomycin and mixing thoroughly—were introduced into the Pyrex bottle. The final solution was labeled as modified Diamond medium. Then, a drop of the vaginal washout was inoculated into a culture tube containing Diamond TYM medium, which was then sealed and incubated at 37°C. Daily microscopic examinations were performed to check for the presence of *T. vaginalis* trophozoites. This process continued for up to 7 days. Specimens that did not show any trophozoites under the microscope by the end of this period were considered negative for *T. vaginalis* using the culture method (Garcia and Alderete, 2007; García, 2016).

### 2.7 Molecular detection of *Trichomonas vaginalis*

Extraction of DNA was performed from 100 μL of vaginal washout samples tested positive by culture methods using the MagMAX™ CORE Nucleic Acid Purification Kit (Cat. No. A32700, Thermo Fisher Scientific, United States). Nested PCR was performed using outer primers Tv8S and Tv9R and inner primers Tv10S and Tv11R (see Table 2), targeting the *T. vaginalis* actin gene (Crucitti et al., 2008; Crucitti et al., 2003). The outer primers targeted amplification of a 1,260 bp fragment, while the inner primers amplified a 1,100 bp fragment. The PCR reaction mixture (25 μL) included 12.5 μL of 2x MyTaq™ Red Mix Master Mix (Cat. No. BIO-25043, Meridian Bioscience, United Kingdom), 0.75 μL (10 μM) of each primer, 5 μL of the DNA template, and 6 μL of deionized water. PCR amplification was performed in two stages using a SimpliAmp™ Thermal Cycler (Cat. No. A24811, Applied Biosystems, United States). The first stage consisted of an initial denaturation step at 95°C for 3 min, followed by 40 cycles of denaturation at 95°C for 30 s, annealing at 55°C for 30 s, and extension at 72°C for 1 min, followed by a final extension at 72°C for 10 min. In the second stage, the process began with an initial denaturation at 95°C for 3 min, followed by 40 cycles of denaturation at 95°C for 30 s, annealing at 50°C for 30 s, and extension at 72°C for 1 min, finishing with a final extension at 72°C for 10 min. After amplification, 7 μL of the PCR product was analyzed via electrophoresis on a 1.5% agarose gel in Tris-acetate-EDTA (TAE) buffer (pH 8.5) and visualized using the InGenius3 gel documentation system (Syngene Bio Imaging, United Kingdom) with 0.5 μg/mL ethidium bromide (Cat. No. E1510, Sigma-Aldrich, Darmstadt, Germany).

**Table 2:** Oligonucleotides were used in Nested PCR for the molecular identification of the *T. vaginalis* in this study.

Name	Description	Sequence (5′–3′)	Reference
*Tv8S*	F1	TCTGGAATGGCTGAAGAAGACG	Crucitti et al. (2008) and Crucitti et al. (2003)
*Tv9R*	R1	CAGGGTACATCGTATTGGTC	
*Tv10S*	F2	CAGACACTCGTTATCG	
*Tv11R*	R2	CGGTGAACGATGGATG	

F1: Forward primer for the actin gene of *T. vaginalis* in the first stage. R1: Reverse primer for the actin gene of *T. vaginalis* in the first stage.

F2: Forward primer for the actin gene of *T. vaginalis* in the second stage. R2: Reverse primer for the actin gene of *T. vaginalis* in the second stage.

### 2.8 Genotyping by PCR-RFLP

The *β*-actin gene was amplified using standard PCR protocols as previously described (Crucitti et al., 2008). Following amplification, the PCR products, which had a target length of 1,100 bp, were digested with three restriction enzymes. Each digestion reaction was prepared according to the manufacturer’s instructions for the specific enzyme. Ten microliters of the amplified product were treated with 0.5 μL of each restriction enzyme: HindII (10 U/μl, 500 units, Cat. No. RE1274), MseI (10 U/μl, 300 units, Cat. No. RE1350), and RsaI (10 U/μl, 1,000 units, Cat. No. RE1324) (Vivantis, Malaysia). Reactions were incubated for 4 h at 37°C for HindII and RsaI, and at 65°C for MseI. The resulting RFLPs were analyzed by electrophoresis on 2.5–3% (w/v) agarose gels. The gels were then visualized and documented using the InGenius3 gel documentation system (Syngene Bio Imaging, United Kingdom). Fragment sizes were determined using the VC 100 bp Plus DNA Ladder (Cat. No. NL1407, Vivantis, Malaysia). The actin genotypes, fragment lengths, and pattern groupings of the *T. vaginalis* isolates are detailed in Table 3.

**Table 3:** Size of fragments, pattern groups and actin genotypes of the *T. vaginalis* (Crucitti et al., 2008).

Genotype	Restriction with HindII (bp)	Restriction with RsaI (bp)	Restriction with MseI (bp)
A	827, 213, 60	568, 236, 190, 106	581, 519
E	827, 213, 60	568, 236, 106, 103, 87	581, 315, 204
G	426, 401, 213, 60	568, 236, 190, 106	581, 519
H	426, 401, 213, 60	568, 236, 106, 103, 87	581, 519
I	426, 401, 213, 60	452, 236, 190, 116, 106	581, 519
M	426, 401, 213, 60	568, 236, 190, 106	581, 333, 186
N	426, 401, 213, 60	568, 236, 106, 103, 87	581, 333, 186
P	426, 401, 213, 60	452, 236, 116, 106, 103, 87	581, 333, 186

### 2.9 Statistical analysis

Data were systematically organized and analyzed using SPSS version 16 (SPSS Inc., Chicago, IL). Results were presented as counts and percentages. To assess statistical significance, the chi-square test (X^2^), also known as Fisher’s exact test, was employed. The student’s *t*-test was used to calculate confidence intervals for the detection rate. A result was considered statistically significant if the two-sided *p* value was less than 0.05.

## 3 Results

### 3.1 Wet-mount microscopic examination and culture

Overall, 8.0% [12/150; 95% Confidence Interval (CI): 3.66–12.34] of female patients with suggestive clinical symptoms tested positive for *T. vaginalis* through microscopy (Supplementary Table S1). On the other hand, *T. vaginalis* trophozoites were isolated from 10.67% [16/150; 95% Confidence Interval (CI): 5.73–15.61] of washout samples from female patients with suggestive clinical symptoms, using cultivation on TYM Diamond’s medium (Supplementary Table S1). The morphology of *T. vaginalis* trophozoites was observed using wet mount microscopic analysis using an oil immersion lens (100x) and following culture on TYM Diamond’s media. The main indicator of a trophozoite’s presence is its distinctive motility, particularly observed through wet mount analysis. Under the microscope, these trophozoites display a nucleus and can take on various shapes, including pear, spherical, or oval. They possess five flagella, with four located in the anterior region and the fifth integrated into the undulating membrane. Regarding motility, trophozoites demonstrate more vigorous movement in TYM Diamond’s medium than in vaginal wet mount samples, where their movement can be influenced by the dynamics of the surrounding fluids and the presence of various components in the sample.

### 3.2 Molecular detection of *Trichomonas vaginalis*

PCR-RFLP analysis of the 16 culture-positive samples revealed that 13 samples were confirmed positive using this molecular method (Table 3). The amplified DNA products were digested by the HindII restriction enzyme, resulting in three distinct fragments: 827 bp, 213 bp, and 60 bp. Further digestion with RsaI yielded five fragments, approximately 87 bp, 103–106 bp, 236 bp, and 568 bp in size. MseI digestion of the amplified product produced three fragments measuring 204 bp, 315 bp, and 581 bp. Analysis of the DNA fragment patterns across all isolates consistently revealed the presence of actin genotype E. Notably, none of the isolates exhibited the actin genotypes A, G, I, M, N, or P. Nested PCR amplification of the *T. vaginalis* actin gene revealed a 1,100 bp fragment in all isolates. Agarose gel electrophoresis confirmed that the length of the amplicons was consistent across all samples, showing no variations.

## 4 Discussion

Trichomoniasis remains one of the most widespread protozoans sexually transmitted infections globally, affecting individuals across all age groups, ethnicities, and socioeconomic backgrounds (Schumann and Plasner, 2024). Rapid and sensitive diagnostic methods are crucial for timely treatment, which helps prevent the transmission of the infection (Shipitsyna et al., 2013) and protects against adverse gynecological and obstetric outcomes (Muzny et al., 2012). In this study, *T. vaginalis* infection was identified in 12 cases (8%) using wet mount preparation of vaginal washouts. This detection rate is lower than the 12.7% positive cases observed through wet microscopic analysis of vaginal swabs from the same cohort. This variation in detection rates is consistent with previous study at Aswan University Hospital outpatient clinic (Hassan et al., 2019) reported a 5% prevalence (10/200) using wet mount techniques, while Mahmoud et al. (2015) found a lower prevalence of 1% (10/1000) at Kasr Al-Ainy Cairo University Hospitals. In our study, *T. vaginalis* was cultured using TYM Diamond’s medium in 150 cases and identified higher detection rate of 10.6% (16 cases) for *T. vaginalis*. Our findings are consistent with those of a previous work (Al-Saeed, 2011) reported a 5.4% detection rate through culture compared to 2.4% with the wet mount method. Similar detection rates were also found in studies by Matini et al. (2012) and Farouk et al. (2021), which reported rates of 1.7 and 2.1%, respectively. Notably, after cultivation, trophozoites of the parasite exhibited significant morphological and motility changes compared to those initially observed through wet mount preparation. These changes included the trophozoites becoming rounded, losing their flagella, and displaying a much slower, barely noticeable motility.

In our study, of the 16 samples that tested positive in culture, 13 yielded results when analyzed using PCR-RFLP. The amplified DNA products from these samples were successfully digested by the restriction enzyme HindII producing three fragments of 60, 213, and 827 bp. Similarly, digestion with RsaI produced five fragments measuring 87, 103, 106, 236, and 568 bp, while digestion with MseI resulted in three fragments of 204, 315, and 581 bp. These DNA fragment patterns were consistent with the presence of the actin genotype E in all the isolates tested. Notably, genotypes A, G, I, M, and P were not detected in any of the samples. The present findings are consistent with several previous molecular studies that reported genotypes G and E as highly prevalent in the African continent. Notably, research on female populations in Kenya and the Democratic Republic of Congo revealed that genotype E was the most common strain of *T. vaginalis*, identified through actin gene analysis, while genotype G was most prevalent in Zambia (Crucitti et al., 2008; Masha et al., 2017). The present finding contrasts with a study (Chetty et al., 2020) reported that PCR-RFLP analysis of the actin gene identified three distinct *T. vaginalis* genotypes, with genotype G being the most prevalent. In research conducted outside of Africa, Momeni et al. (2015) identified five distinct *T. vaginalis* genotypes, with genotype G being the most prevalent among both men and women in Iran. However, more recent studies (Khalili et al., 2017; Tavakoli Oliaee et al., 2017; Matini et al., 2012) revealed different genotype distributions compared to those reported by Momeni et al. (2015) and the patterns observed in Africa (Crucitti et al., 2008; Masha et al., 2017). In this context a previous work (Matini et al., 2012) examined symptomatic and asymptomatic women visiting gynaecology clinics in western Iran and found genotype A to be the most dominant based on actin gene digestion profiles. Similarly, several previous works conducted in Iran reported that genotype H was the most frequent among women attending general healthcare services (Khalili et al., 2017; Tavakoli Oliaee et al., 2017). This diversity indicates potential variability in the genetic makeup of *T. vaginalis* in certain populations. The genetic differences observed in these studies might be explained by factors such as larger sample sizes or sampling from regions with higher infection rates (Abou-kamar et al., 2017).

## 5 Conclusion

Our study showed that the culture method for detecting *T. vaginalis* seems to be more sensitive than direct microscopic examination. Phosphate-buffered vaginal washout samples were utilized for direct wet mount microscopic examination, with the goal of rapidly detecting motile *T. vaginalis* trophozoites by identifying their distinctive flagellar movement. Additionally, our genetic analysis confirmed the molecular identification of *T. vaginalis* in Upper Egypt, indicating that only genotype E was present in this study. Collectively, these findings suggest that while culture techniques and specimen collection methods are crucial for accurate diagnosis, understanding genetic variations of the parasite is essential for regional epidemiological assessments and targeted interventions. Future research is recommended to investigate the prevalence and molecular characteristics of *T. vaginalis* on a larger scale within the Egyptian population. Additionally, there is a need to raise public health awareness about the importance of adopting strict hygiene measures to control the spread of this sexually transmitted infection.

## Data Availability

The original contributions presented in the study are included in the article/Supplementary material, further inquiries can be directed to the corresponding author.
