# Supplementary material for: Microscopic and molecular detection of Trichomonas vaginalis in outpatients seeking medical care in Upper Egypt
Source: Front Microbiol. 2024 Nov 20;15:1499270. doi: 10.3389/fmicb.2024.1499270 (PMC11615069; doi:10.3389/fmicb.2024.1499270)
Supplement: Supplementary file 1 [file Table_1.DOCX]

| **Sample ID** | **Age (yrs.)** | **Wet mount** | **Culture media** | **PCR-RFLP** |
| --- | --- | --- | --- | --- |
| 2 | 40 | +ve | +ve | +ve |
| 5 | 22 | +ve | +ve | +ve |
| 9 | 27 | - ve | +ve | - ve |
| 10 | 31 | +ve | +ve | +ve |
| 17 | 39 | - ve | +ve | +ve |
| 25 | 15 | +ve | +ve | +ve |
| 27 | 32 | +ve | +ve | +ve |
| 32 | 18 | +ve | +ve | +ve |
| 36 | 25 | - ve | +ve | - ve |
| 42 | 37 | +ve | +ve | +ve |
| 57 | 23 | +ve | +ve | +ve |
| 94 | 19 | - ve | +ve | - ve |
| 112 | 26 | +ve | +ve | +ve |
| 125 | 18 | +ve | +ve | +ve |
| 135 | 22 | +ve | +ve | +ve |
| 147 | 35 | +ve | +ve | +ve |

**Supplementary Table 1**. The distribution pattern of positive *Trichomonas vaginalis* cases identified in this study was investigated using three diagnostic methods: Wet mount microscopy, culture on TYM Diamond’s medium, and PCR amplification followed by restriction enzyme digestion (PCR-RFLP). The results are presented with the sample ID, age, and the method used.

-ve: negative; +ve: positive
